# Intestinal stenosis of Garré following emergency ventral hernia repair

**DOI:** 10.1002/ccr3.4558

**Published:** 2021-08-15

**Authors:** Bethany Padgett, Deborah Gurung, Charalampos Seretis, Lourdusamy Selvam

**Affiliations:** ^1^ Department of General Surgery George Eliot Hospital NHS Trust. Address: College Street Nuneaton UK

**Keywords:** emergency, Garré, hernia, stenosis, surgery

## Abstract

Intestinal stenosis of Garré can occur as a result of prolonged ischemia after all types of hernia surgery.

## Graphical abstract

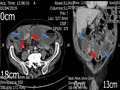

## 1 CASE DESCRIPTION

Intestinal stenosis of Garré is a rare complication following emergency hernia repairs, described mainly after inguinal or femoral hernia surgery. Herein, we present the first reported case of symptomatic intestinal stenosis of Garré occurring after emergency primary ventral hernia repair.

**FIGURE 1:**
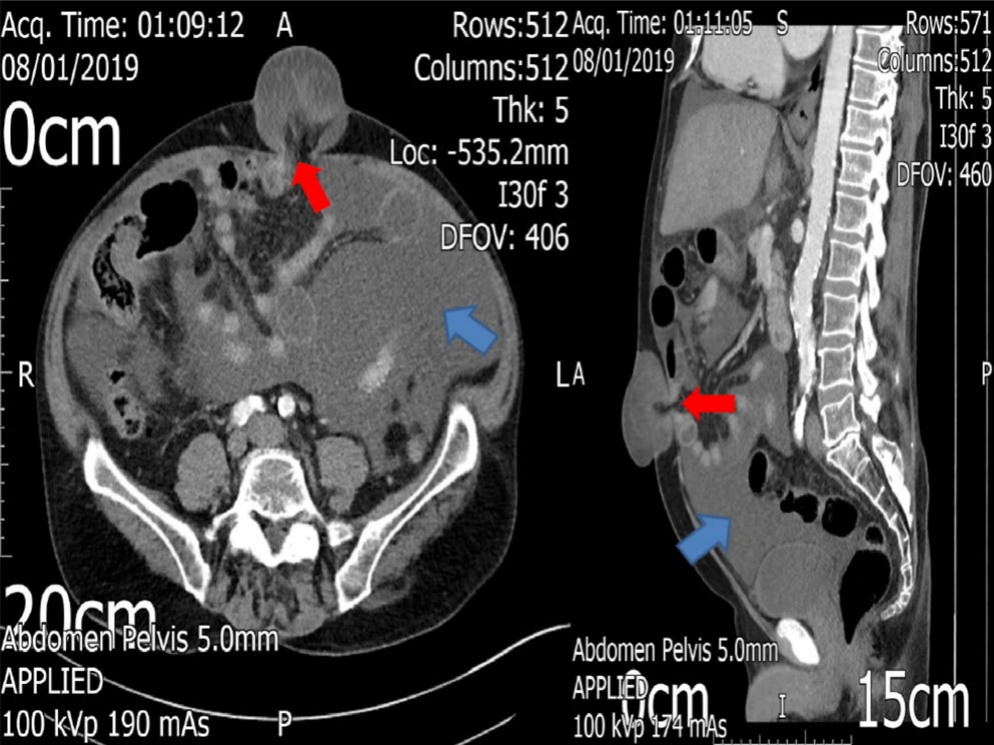
Axial and sagittal views of the CT scan prior to the initial emergency ventral hernia repair, showing the site of the incarcerated umbilical hernia, with congested small bowel protruding through the defect. Note the compression of the small bowel at the level of the constriction ring of the hernia, suggesting the development of closed‐loop obstruction (red arrows) and the presence of large‐volume, tense cardiac ascites (blue arrows)

A 63‐year‐old Caucasian man was admitted with symptoms of chronic intestinal obstruction. He had previously undergone an emergency suture repair of a long‐standing umbilical hernia. Prior to that procedure, a computed tomography (CT) scan demonstrated the presence of a small bowel loop in the umbilical hernia defect (Figure 1). Intraoperatively, a segment of small bowel was found entrapped within the hernia sac, with features of evolving hemorrhagic necrosis; however, it was deemed salvageable. Unfortunately, for the following three months the patient had ongoing symptoms of chronic subacute intestinal obstruction.

**FIGURE 2:**
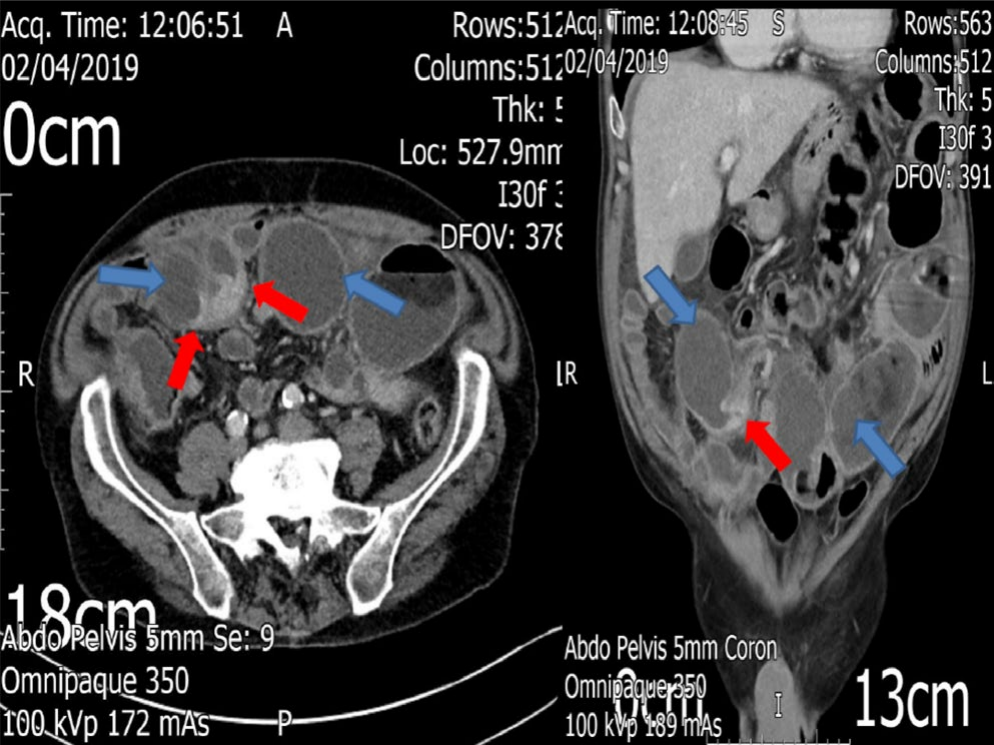
Axial and coronal views of the CT scan prior to the second operation; note the presence of the stenosis of Garré in the previously herniated small bowel segment, with fibrotic appearances and luminal narrowing (red arrows), causing gross prestenotic small bowel dilatation (blue arrows)

Upon readmission, a repeat CT scan of his abdomen and pelvis showed features suggestive of gastrointestinal obstruction due to segmental small bowel stricture (Figure 2). The patient was scheduled for emergency laparotomy; a 5‐cm structuring ileal segment was identified and resected. This type of intestinal stricture has been reported as a rare complication following emergency hernia repairs, in which the incarcerated small bowel segment is deemed macroscopically viable but has already sustained prolonged venous stasis, accompanied by a degree of irreversible, nontransmural ischemic changes. The above‐mentioned pathophysiological changes can lead to the development of hypoxia‐related fibrosis, and eventually strictures. This rare complication more commonly occurs postemergency right‐sided inguinal and femoral hernia repairs,^1^, ^2^ in which the naturally narrower distal ileum can herniate; to the best of our knowledge, our presented case is the first in the international literature to report the occurrence of intestinal stenosis of Garré following the repair of a primary ventral hernia.

## CONFLICTS OF INTEREST

The authors declared no potential conflicts of interest with respect to the research, authorship, and/or publication of this article.

## AUTHOR CONTRIBUTIONS

BP and DG: contributed to the clinical data collection and prepared the case report. CS and LS: contributed to the design of the case report presentation and performed the final revision of the manuscript.

## ETHICAL STATEMENT

Informed consent was obtained from the patient and is available upon request by the editorial office; no ethical committee approval was required for the publication of this case report.

## Data Availability

The presented data are part of the manuscript.
